# Evolutionary origin of alpha rhythms in vertebrates

**DOI:** 10.3389/fnbeh.2024.1384340

**Published:** 2024-04-08

**Authors:** Takashi Shibata, Noriaki Hattori, Hisao Nishijo, Satoshi Kuroda, Kaoru Takakusaki

**Affiliations:** ^1^Department of Neurosurgery, Toyama University Hospital, Toyama, Japan; ^2^Department of Neurosurgery, Toyama Nishi General Hospital, Toyama, Japan; ^3^Department of Rehabilitation, Toyama University Hospital, Toyama, Japan; ^4^Faculty of Human Sciences, University of East Asia, Yamaguchi, Japan; ^5^The Research Center for Brain Function and Medical Engineering, Asahikawa Medical University, Asahikawa, Japan

**Keywords:** alpha rhythms, pedunculopontine nucleus, vertebrates, nocturnal mammals, pallium, neocortex

## Abstract

The purpose of this review extends beyond the traditional triune brain model, aiming to elucidate the evolutionary aspects of alpha rhythms in vertebrates. The forebrain, comprising the telencephalon (pallium) and diencephalon (thalamus, hypothalamus), is a common feature in the brains of all vertebrates. In mammals, evolution has prioritized the development of the forebrain, especially the neocortex, over the midbrain (mesencephalon) optic tectum, which serves as the prototype for the visual brain. This evolution enables mammals to process visual information in the retina-thalamus (lateral geniculate nucleus)-occipital cortex pathway. The origin of posterior-dominant alpha rhythms observed in mammals in quiet and dark environments is not solely attributed to cholinergic pontine nuclei cells functioning as a 10 Hz pacemaker in the brainstem. It also involves the ability of the neocortex’s cortical layers to generate traveling waves of alpha rhythms with waxing and waning characteristics. The utilization of alpha rhythms might have facilitated the shift of attention from external visual inputs to internal cognitive processes as an adaptation to thrive in dark environments. The evolution of alpha rhythms might trace back to the dinosaur era, suggesting that enhanced cortical connectivity linked to alpha bands could have facilitated the development of nocturnal awakening in the ancestors of mammals. In fishes, reptiles, and birds, the pallium lacks a cortical layer. However, there is a lack of research clearly observing dominant alpha rhythms in the pallium or organized nuclear structures in fishes, reptiles, or birds. Through convergent evolution, the pallium of birds, which exhibits cortex-like fiber architecture, has not only acquired advanced cognitive and motor abilities but also the capability to generate low-frequency oscillations (4-25 Hz) resembling alpha rhythms. This suggests that the origins of alpha rhythms might lie in the pallium of a common ancestor of birds and mammals.

## Introduction

The concept of the “Triune Brain,” proposed by Dr. Paul MacLean, suggests the evolution of the complex human brain through the gradual addition of new regions to the simpler brains of our ancestors (MacLean, 1990). However, recent advances in comparative neuroscience have revealed that vertebrates, including fishes, reptiles, and birds, also possess regions similar to the gray matter of the cerebral cortex (Jarvis et al., 2005). The brains of vertebrates initially develop from a common neural tube, where neural stem cells are directed to specialize into species-specific brain structures through internal molecular and cellular signaling pathways governed by genes. This specialization leads to the formation of the forebrain, midbrain, and hindbrain. Additionally, these three segments extensively expand dorsally as extensions of gray matter, giving rise to the cerebrum, the roof or tectum of the midbrain, and cerebellum, respectively.

**Figure 1:**
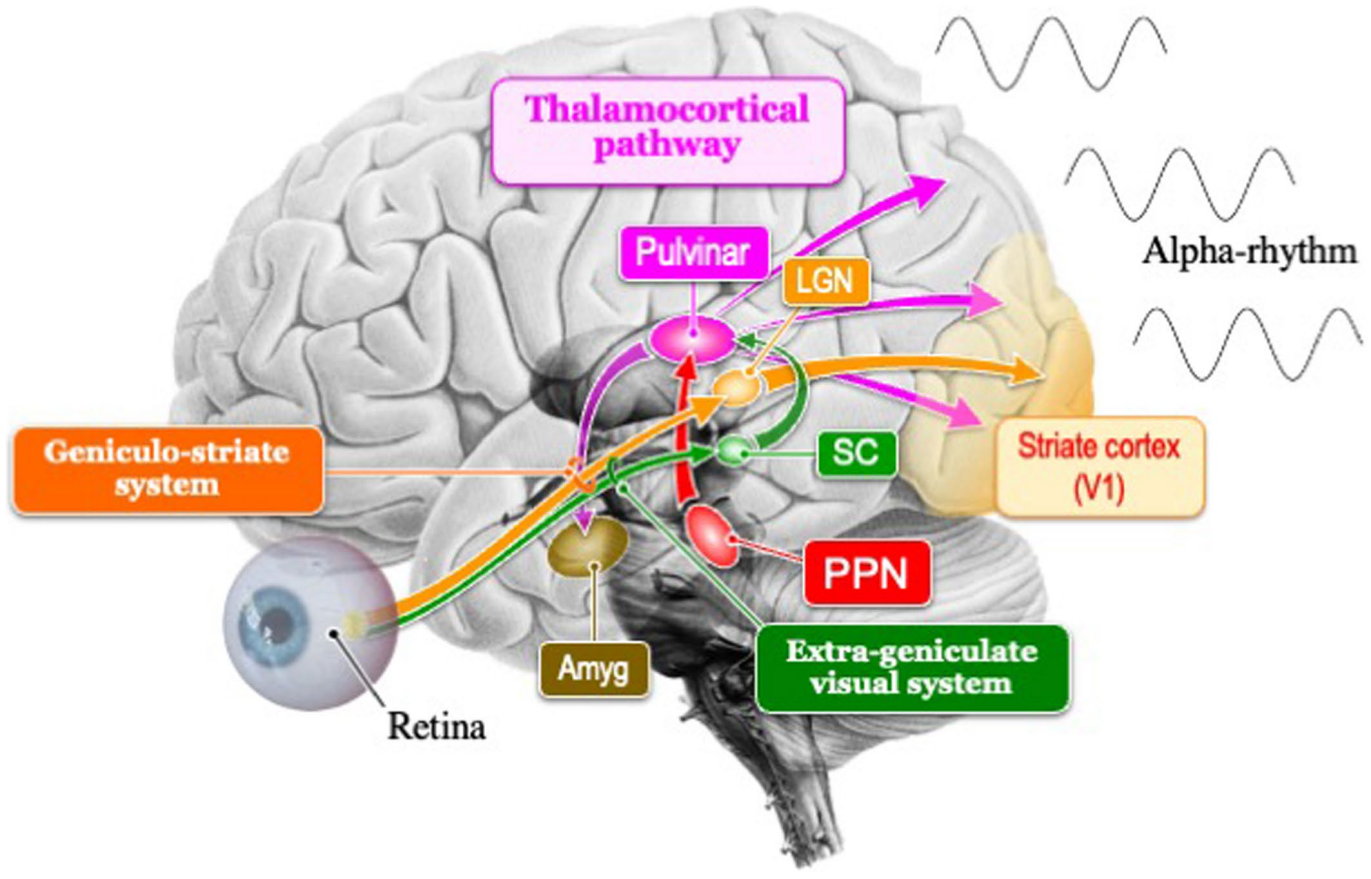
Schema of the two visual pathways (extrageniculate visual system and geniculo-striate system) and thalamocortical pathway that generates the posterior dominant alpha rhythm. In mammals, the geniculo-striate system has evolved dominantly over the extrageniculate visual system. SC, superior colliculus; Amg, amygdala; LGN, lateral geniculate nucleus; PPN, Pedunculopontine nucleus.

In the context of evolutionary visual information processing, the subcortical visual system (extrageniculate system), which encompasses the midbrain, is traditionally regarded as ancient, while the cortical visual system (geniculo-striate system), including the forebrain, is seen as more recent. However, recent findings suggest that even in primitive vertebrates like lampreys, cortical visual pathways might exist (Isa et al., 2021). This suggests that the genetic blueprint for both visual pathways might have been present in all vertebrates from the beginning. Essentially, the genetic information guiding the development of the posterior cortex in mammals, within these two visual pathways, could influence the dominance of the occipital alpha rhythm (Figure 1).

The evolution of the midbrain (mesencephalon) in fishes, reptiles, and birds that operate in bright environments is primarily influenced by visual information. In contrast, the ancestors of nocturnal mammals, which evolved in dark environments, underwent evolution not only in the midbrain but also enveloped the thalamus and neocortex. These adaptations were crucial for their survival, as the ancestors of nocturnal mammals primarily engaged in nocturnal activities to compete with dinosaurs for survival. However, following the extinction of dinosaurs, while some mammals retained their nocturnal habits, others emerged that transitioned from nocturnal to diurnal behavior (Maor et al., 2017).

Humans have been observed to increase alpha power and peak frequency to adapt to dark environments (Webster and Ro, 2020). The strength of cortical connectivity (triangle-based clustering) in the alpha band is believed to contribute to the promotion of arousal from coma (Bareham et al., 2020). Additionally, visual stimuli have been found to suppress alpha activity, whereas internal processes such as mental calculation and working memory enhance it (Cooper et al., 2003; Palva and Palva, 2007). These findings relate to the physiological adaptation of unique alpha rhythms in mammals, but the evolutionary processes explaining why alpha rhythms acquired such physiological mechanisms have not been fully explored. Therefore, based on the findings presented above, we hypothesize that the unique alpha rhythms of mammals might have supported their nocturnal behavior, especially concerning the role this rhythm has on arousal and internal cognitive processes. This review delves into the neurodevelopmental processes of vertebrates in evolution, comprehensively investigates existing literature, and summarizes the origin and evolutionary significance of alpha rhythms in vertebrates (Table 1).

**Table 1:** Summary of brain evolution associated with EEG rhythms.

Vertebrate	Fishes	Reptiles	Birds	Mammals
Characteristics of telencephalon	Pallium			Neocortex
	Primitive layered structure*			Sophisticated layered structure
Nuclear structures	Organized nuclear structure		Highly organized nuclear structure*	
Two visual systems	Extrageniculate system			Geniculo-striate system
Hindbrain	Pedunculopontine nucleus (10 Hz pacemakaer cells**)			
Lifestyle	Mainly diurnal			Primarily nocturnal
Cognitive ability	Fundamental ability		Advanced ability***	
Dominant rhythmic EEG	Without reporting in the literature		Low-frequency oscillations	Posterior dominance alpha rhythms
Characteristics of alpha rhythms	Without reporting in the literature			Traveling waves along the layers, and waxing and waning patterns

*In the avian Wulst, there are structurally various layers that resemble the neocortex of mammals. Additionally, avian sensory DVRs have hierarchically organized circuits. **10 Hz pacemaker cells in the PPN have only been reported in mammals. ***Cognitive abilities in primates have evolved dramatically.

## Evolution of the vertebrate brain

The neural tube, consisting of three parts known as the forebrain, midbrain, and hindbrain, ultimately develops into five distinct regions of the brain: telencephalon, diencephalon, mesencephalon, metencephalon, and myelencephalon. While the vertebrate brain universally follows a consistent pattern, the midbrain in mammals has evolved along a different trajectory compared to fishes, reptiles, and birds. In fishes, reptiles, and birds, a portion of the midbrain transforms into the optic lobe for visual information processing. Conversely, in mammals, a part of the forebrain has evolved into the neocortex. Thus, the evolution of visual information processing in the midbrain, primarily dependent on the optic tectum in fishes, reptiles, and birds, contrasts with mammals, which evolved by incorporating the posterior cortex, a part of the forebrain.

During evolution, the mammalian neocortex developed a mechanism for the migration of new neurons, leading to the formation of a distinctive six-layered structure absent in fishes, reptiles, or birds (Suzuki et al., 2012; Ohtaka-Maruyama et al., 2018). While it is well established that alpha oscillations occur in the mammalian neocortex under dark and quiet conditions as a physiological phenomenon, the evolutionary adaptation whereby only the six-layered neocortical structure of mammals acquired mammalian-specific alpha rhythms remains poorly understood. In contrast, reptiles internally possess a primitive-layered pallium (i.e., a three-layered structure) similar to the mammalian hippocampus and olfactory cortex (Aboitiz et al., 2003), which might generate theta band rhythms. Furthermore, due to the lack of complex connections between the three different brain regions found in mammals—namely the neocortex (part of the forebrain), the thalamus (part of the diencephalon), and the midbrain—it might be difficult for reptiles to generate alpha oscillations equivalent to those found in mammals. However, since alpha rhythms might emerge in non-laminar nuclear organization such as the pedunculopontine nucleus (PPN), it is necessary to explore traces of alpha rhythms in these structures. In the future, scientific investigation will be required to examine the differences between two potential mechanisms for alpha rhythm generation: one originating from the uniquely developed alpha rhythms in the mammalian neocortex and the other from nuclear organization common to vertebrates, suggesting the presence of two distinct mechanisms for alpha rhythm generation.

The commonality in the midbrain nucleus structures among vertebrates, such as dopamine neurons in the substantia nigra, noradrenaline neurons in the locus coeruleus, and acetylcholine neurons in the pedunculopontine nucleus (PPN), raises the possibility that traces of alpha rhythm generation exist in fishes, reptiles, and birds. For example, the locus coeruleus in mammals exhibits a highly refined function with abundant neurons (3,000 in rats, 7,000 in monkeys, up to 50,000 in humans) compared to the primitive nature and limited number (10–20) in fishes (Mouton et al., 1994; Holzschuh et al., 2001; Sharma et al., 2010; Farrar et al., 2018; Wang et al., 2022). When considering the possibility that, similar to the locus coeruleus, the number of PPN neurons might be low, it might be insufficient to generate alpha rhythms in the PPN of fishes and reptiles.

## 10 Hz pacemaker cells in the PPN

The PPN, located in the dorsal tegmentum of the brainstem, is highly conserved across species and is known to be involved in the control of various functions such as movement, reward, motivation, arousal, and behavioral states (Benarroch, 2013). Takakusaki reported that in the mammalian PPN, neurons spontaneously fire rhythmically at an average of approximately 10 Hz (Takakusaki and Kitai, 1997). These PPN neurons not only exhibit a baseline firing frequency of 8 to 10 Hz during quiet wakefulness without external input (Simon et al., 2010; Kezunovic et al., 2013) but are also known to maximally fire at gamma frequencies when appropriately stimulated electrically (Simon et al., 2010; Garcia-Rill et al., 2011).

This firing characteristic is most active during wakefulness, functioning as part of the ascending reticular activating system and contributing to the transition of overall brain activity from sleep to wakefulness. In other words, the sustained discharge occurring at approximately 10 Hz within the PPN contributes to maintaining the awake state, promoting increased cortical activity through rhythmic patterns of sleep and wakefulness in vertebrates, and enhancing the awake state (Petzold et al., 2015).

In mammals, the PPN plays a central role not only in regulating the rhythm of sleep and wakefulness but also in modulating the flexibility of behavior through cholinergic modulation (Mena-Segovia and Bolam, 2017). Consistency between the PPN and cortical activity in the alpha band is particularly pronounced during walking stages, especially when the stepping rhythm is regular (He et al., 2021). This suggests that the PPN might have played a crucial role in ancestral mammals walking rhythmically and regularly in the dark.

Cholinergic neurons in the PPN, common in vertebrates, may be the origin of the 10 Hz frequency of alpha rhythms. The rhythmicity of the alpha rhythm in mammals is created in the thalamic pulvinar, and the membrane potential in the thalamic neurons is controlled by the PPN that regulate the arousal level (Steriade, 2000). These neurons could influence cortical activity through input to the thalamus, supporting the sleep–wake cycle and behavioral arousal in nocturnal mammals (Figure 1). The 10 Hz frequency of alpha rhythms, emanating from the PPN which governs transitions between sleep and wakefulness, facilitates shifts to higher or lower frequencies. The frequency pattern of PPN transitions from active wakefulness to relaxed wakefulness depends on the state of wakefulness. Active wakefulness involves low beta band (12–18 Hz) activity driven by top-down control and feedback from the external environment, while relaxed wakefulness, typically occurring in quiet nighttime environments, is characterized by activity in the alpha band (8–12 Hz).

## Two visual systems in mammals

In mammals, there are two visual pathways (Figure 1): the cortical visual pathway, which transmits visual information from the retina through the lateral geniculate nucleus to the primary visual cortex (cortical visual pathway), and the subcortical visual pathway, directly inputting visual information to the superior colliculus of the midbrain and pulvinar of thalamus (Nguyen et al., 2013; Le et al., 2019). The subcortical visual pathway (retina → superior colliculus → thalamus → amygdala) begins its activity in the early stages of infancy in humans, playing a crucial role in rapidly detecting visually important information for survival and social behavior (Striano and Reid, 2006). Afterward, it has been observed that the cortical visual pathways mature during the late infancy period (8–12 years old), leading to the development of alpha rhythms within the frequency range of adults (Anderson and Perone, 2018). Recognizing the sensitive period for the plasticity of this alpha activity, it has been revealed that visual experiences between the ages of 3–6 are crucial (Campus et al., 2021). It has been demonstrated that not only do visual input restrictions such as darkness or closing one’s eyes (Webster and Ro, 2020), but also instances where mothers read picture books to their children (Hasegawa et al., 2021), trigger alpha activity. Interestingly, Hasegawa suggests that such interactions between infants and intimate person lead to an increase in high local segregation with high global integration of alpha-band networks, resulting in the promotion of alpha rhythms in concentrated and relaxed states of arousal.

Alpha blocking is a well-known physiological phenomenon characterized by the presence of low-amplitude irregular fast waves upon opening the eyes. This occurs due to the suppression of alpha rhythms caused by inputs from visual field to the visual cortex from open eyes. The alpha blocking can be explained by an increase in external inputs to inhibitory neurons in the cerebral cortex (Hartoyo et al., 2020). Investigating the physiological phenomena of alpha blocking in the future will help us understand why mammals have evolved two distinct modes of visual processing: open-eyed and closed-eyed. This exploration is crucial for gaining insights into the reasons behind this duality in the mammalian neocortex.

The mammalian neocortex comprises layers running parallel to the cortical surface and columns running perpendicular to these layers. This arrangement forms cylindrical and laminar structures with orthogonal fibers arranged both radially and tangentially. Each layer in the laminar structure consists of granular components, including the granular layer IV, the supragranular layers II/III, and the infragranular layers V/VI. Notably, in primates, the neocortex has evolved by significantly enlarging the supragranular layers during the later stages of neural development (Hill and Walsh, 2005). Visual information entering the neocortex flows from the granular layer to the supragranular layer and then to the infragranular layer. However, in memory information, primates have evolved to reverse this flow of signals, moving from the infragranular layer to the supragranular layer (Takeuchi et al., 2011).

The question of whether alpha rhythms evolved independently in the newly evolved neocortex of mammals remains unresolved. Birds and mammals have both independently evolved similar brain structures that provide comparable sensory-motor and cognitive abilities, a phenomenon known as convergent evolution (Veit and Nieder, 2013; Ditz and Nieder, 2015). Despite their primitive layered structure, birds not only demonstrate remarkable cognitive and motor abilities comparable to mammals (Guy et al., 2017), but also reportedly generate low-frequency rhythms (4-25Hz) similar to mammalian alpha rhythms (Hahn et al., 2022). Therefore, it is necessary to investigate the differences between the alpha rhythms present in mammals, with a peak frequency of 10 Hz, and the alpha-like low-frequency oscillations in birds. Interestingly, the frequency of primary rhythms in the visual cortex of mammals correlates with brain size, ranging from 3-6 Hz in mice (Senzai, et al., 2019), 6-12 Hz in cats (Lörincz et al., 2009), to 8-12 Hz in humans. Thus, the emergence of the primate-specific alpha rhythm at 10 Hz might be associated with both the enlargement of the brain and the maturation of circuits in the neocortex and pulvinar (Cortes, et al., 2021).

## Alpha rhythms in the mammalian neocortex and avian pallium

Mammals, including humans, possess a neocortex characterized by a six-layered structure (Hill and Walsh, 2005). The development of the layer IV in sensory areas, such as somatosensory, visual, and auditory regions, is notably influenced by thalamic cortical axons. Unlike motor areas, which lack thalamic inputs, these sensory regions receive input from the thalamus (Sato et al., 2012, 2022). The visual field, auditory field, and sensory field, each receiving inputs from the thalamus, constitute distinct brain functional networks formed by oscillatory components in the alpha band (Hipp et al., 2012). In studies involving rat brain tissue, the role of pyramidal neurons in the fourth layer in inducing alpha rhythms in response to gamma stimuli has been proposed (Traub et al., 2020). In this experiment, pharmacologically stimulating the primary visual cortex with gamma rhythms led to the emergence of alpha rhythms. This occurred through the generation of distinct population synchrony bursts, actively suppressing the stimulus response. When transitioning from an open-eye state to closing the eyelids, thereby abruptly interrupting visual information, the results suggest that the mammalian neocortex can autonomously and efficiently generate alpha rhythms.

Natural phenomena, such as avalanches and earthquakes, which deviate from the normal distribution in terms of scale and frequency, are well known to follow a power-law distribution. For instance, “Omori’s law,” which describes the decay of aftershock frequency following a main earthquake according to a power-law as time progresses, is widely recognized (Omori, 1894). In the pallium of zebrafish, the activity of trigger neurons has been reported to initiate large-scale cascades of reactions, leading to the occurrence of neural avalanches (Ponce-Alvarez et al., 2018). Vertebrates are believed to leverage this physiological phenomenon of neural avalanches to enhance intrinsic information processing capabilities in spontaneous brain activity, linking it to the rapid acquisition of food and escape behaviors from predators. The phenomenon of neural avalanches has been observed not only in the pallium of fish brains but also in the human neocortex. It has been revealed that the distinctive waxing and waning phenomenon of alpha rhythms is utilized by neural avalanches (Lombardi et al., 2023). The decay of alpha rhythms suppresses neural activity by transmitting alpha-mediated inhibition in intermittent pulses within a single alpha cycle (approximately 100 msec). On the other hand, excitation is continuously enhanced over several alpha cycles, amplifying neural activity. This unique waxing and waning phenomenon of alpha rhythms is generated in accordance with Omori’s law, which is a power-law (Lombardi et al., 2023).

Simultaneous recordings from the human neocortex and thalamus revealed that during quiet wakefulness, cortical alpha oscillations independently emerged before thalamic alpha oscillations. Furthermore, propagation from higher-order cortical areas to lower-order ones, and from the cortex to the thalamus, was observed (Halgren et al., 2019). In the posterior cortex, alpha oscillations were observed to propagate from higher-order anterosuperior areas to the occipital pole, indicating that cortical alpha oscillations guide thalamic alpha oscillations, particularly from the cortical surface. Additionally, in the primary visual cortex (V1), gamma oscillations initiate in layer IV and propagate through the deep and superficial layers of the cortex, whereas alpha oscillations reportedly start from layers I, II, and V of I1 and propagate toward layer IV (Van Kerkoerle et al., 2014). The propagation of these alpha oscillations differs from the feedforward direction of gamma oscillations, instead propagating in a feedback direction. It is believed that the ability of these alpha oscillations to propagate within the cortex is a prominent feature of the mammalian cortex due to its laminar structure. However, although the avian pallium is structurally simple nuclear organization, a recent study has revealed that both mammalian neocortex and avian pallium possess an orthogonal fiber architecture composed of fibers oriented radially and tangentially (Stacho et al., 2020).

Avian pallium consists of the Wulst and the dorsal ventricular ridge (DVR), both characterized by nuclear organization (Stacho et al., 2020). These structures feature a sensory input zone akin to the granular layer IV. The Wulst integrates somatosensory and visual nuclear organization, while the DVR encompasses nuclear organizations related to somatosensory, trigeminal, visual, and auditory functions. Additionally, orthogonal fibers facilitate functional and structural connections between distant nuclear organizations. However, it remains unclear whether the avian-specific cortical-like fiber structure, which functionally connects various nuclear organizations, can produce prototypes of alpha rhythms within the pallium akin to the laminar structures observed in mammals.

Around 320 million years ago, it is believed that a common ancestor of mammals and birds inhabited the Earth. As evolution progressed, the dorsal pallium of this ancestor underwent independent differentiation into the brains of mammals and birds (Stacho et al., 2020). Hence, it is plausible that the origin of alpha rhythms might be traced back to the dorsal pallium of this common ancestor. This aspect remains a potential subject for future research.

## Mammalian ancestors that awakened during the night

The first mammals to appear between 220 million and 166 million years ago during the Jurassic period are known to have been primarily nocturnal throughout much of the dinosaur era (Maor et al., 2017). The ability of our mammalian ancestors to maintain wakefulness in darkness was crucial for the survival of mammals during the dinosaur era. Mammalian ancestors, in order to avoid the danger of being preyed upon by dinosaurs, spent long hours hiding in darkness. Consequently, the color vision of mammals, unlike that of fishes, reptiles, and birds, diminished in its ability to distinguish colors (Gerl and Morris, 2008). In alpha rhythm activities, the increase in alpha power and peak frequency in darkness conditions not only enhances the identification of faint visual stimuli from the external environment but also facilitates the shift of attention from external inputs to internal cognitive processes (Webster and Ro, 2020). Based on this, it is possible that the ancestors of nocturnal mammals chose a survival strategy of actively suppressing external visual input using alpha rhythms, and reinforcing their memory and recall as internal cognitive processes in darkness.

Nocturnal mammals might have evolved unique cognitive processing abilities to adapt dark environments successfully. In contrast, for diurnal fishes, reptiles, and birds, nighttime activities might face limitations in harnessing alpha rhythms. Specifically, since primates transitioned from nocturnality to diurnality approximately 52 million years ago (Maor et al., 2017), they have developed the ability to suppress distracting visual stimuli from the external environment and enhance the shift of attention toward mental operations, such as mental rotation tasks and visual imagery, using alpha rhythms (Sauseng et al., 2009; Mathewson et al., 2011). Furthermore, it is theorized that humans have developed the ability to recognize past, present, and future time through a clock function using 10 Hz alpha rhythms, facilitated by a brain network centered around the precuneus (Takahashi and Kitazawa et al., 2017).

## Conclusion

During the dinosaur era, it’s possible that ancestors of nocturnal mammals developed enhanced cortico-cortical connections, allowing them to transmit alpha rhythms characterized by traveling waves across cortical layers. Additionally, the evolution of alpha rhythms, which display patterns of waxing and waning, might have facilitated the shift of attention from external visual stimuli to internal cognitive processes as an adaptation to dark environments. This adaptation potentially drove the development of new cognitive functions in the mammalian neocortex. In contrast, while the pallium in fishes, reptiles, and birds has a primitive-layered pallium and organized nuclear structures, there are no documented instances of its interaction with alpha rhythms similar to those found in mammals. However, the presence of PPN’s 10Hz pacemaker cells, and birds, which produce alpha-like low-frequency oscillations, could provide insights into the origins of alpha rhythms.

## Author contributions

TS: Conceptualization, Data curation, Formal analysis, Investigation, Project administration, Writing – original draft. NH: Supervision, Validation, Writing – review & editing. HN: Supervision, Validation, Writing – review & editing. SK: Validation, Writing – review & editing. KT: Supervision, Validation, Visualization, Writing – review & editing.
